# Cross-Regulation of the Cellular Redox System, Oxygen, and Sphingolipid Signalling

**DOI:** 10.3390/metabo13030426

**Published:** 2023-03-14

**Authors:** Andrea Huwiler, Karl-Friedrich Beck, Josef Pfeilschifter

**Affiliations:** 1 Institute of Pharmacology, University of Bern, Inselspital INO-F, CH-3010 Bern, Switzerland; 2 Institute of General Pharmacology and Toxicology, Goethe University Frankfurt am Main, Theodor-Stern Kai 7, D-60590 Frankfurt am Main, Germany

**Keywords:** sphingolipids, redox, reactive oxygen species, nitric oxide, hydrogen sulfide, hypoxia

## Abstract

Redox-active mediators are now appreciated as powerful molecules to regulate cellular dynamics such as viability, proliferation, migration, cell contraction, and relaxation, as well as gene expression under physiological and pathophysiological conditions. These molecules include the various reactive oxygen species (ROS), and the gasotransmitters nitric oxide (NO**∙**), carbon monoxide (CO), and hydrogen sulfide (H_2_S). For each of these molecules, direct targets have been identified which transmit the signal from the cellular redox state to a cellular response. Besides these redox mediators, various sphingolipid species have turned out as highly bioactive with strong signalling potential. Recent data suggest that there is a cross-regulation existing between the redox mediators and sphingolipid molecules that have a fundamental impact on a cell’s fate and organ function. This review will summarize the effects of the different redox-active mediators on sphingolipid signalling and metabolism, and the impact of this cross-talk on pathophysiological processes. The relevance of therapeutic approaches will be highlighted.

## 1. Introduction

The ability to define and address novel problems, especially across disciplines is crucial for the innovations that really trigger eminent advances in science. In this article, we present our current knowledge about the core concepts of bioactive sphingolipids and redox mediators in cellular signalling processes in general and their potential cross-communication in particular. We nowadays understand that lipid and redox signalling is highly regulated and complex, but little is known about the interconnection of both signalling pathways. In the first section, we describe the basics of redox and sphingolipid signalling, before we summarize in a second section what is known about their crosstalk. Exploration of this question is challenging because it refers to chemically and biophysically different kinds of mediators. Moreover, in large parts, the literature on the potential interaction of both classes of mediators is descriptive, an inevitable feature of emerging fields in science. Nevertheless, we trust that this review will help to create a conceptual framework that systematically organizes known data and although it requires much additional work, it finally may make up an attractive concept, especially for translational medicine and drug development.

### 1.1. Redox Signalling

**Figure 1:**
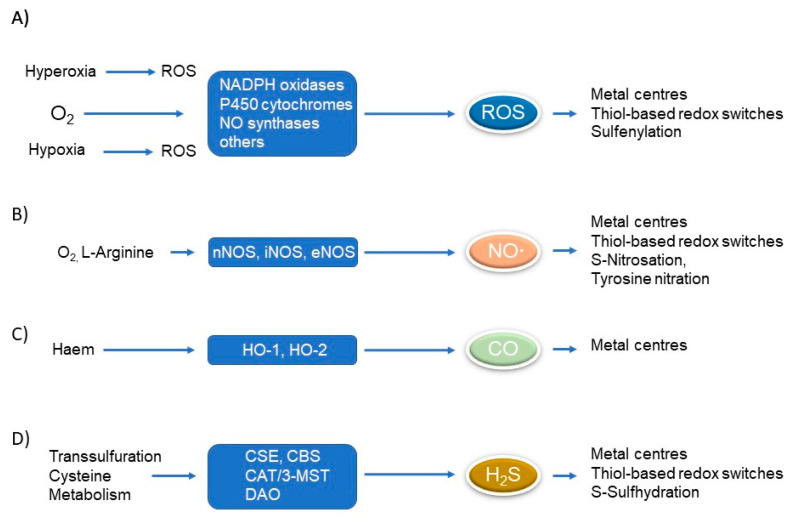
Synthesis and action of redox-mediators in mammalian cells. (**A**) Formation of ROS by the enzymatic activity of several oxidases. Note that enhanced ROS formation occurs during normoxia and is enhanced in a hyperoxic as well as hypoxic environment. (**B**) Generation of NO**∙** by NO synthases. (**C**) Formation of CO by HO-1 and HO-2. (**D**) Synthesis of H_2_S via the transsulfuration pathway and cysteine metabolism. ROS, NO**∙**, and H_2_S potently form thiol-based redox switches on cysteines, and all redox-mediators including CO attack metal centres of proteins. Abbreviations: CAT, cysteine aminotransferase; CBS, cystathionine β-synthase; CSE, cystathionine γ-lyase; CO, carbon monoxide; DAO, D-aminotransferase; H_2_S, hydrogen sulfide; HO, heme oxygenase; 3-MST, 3-mercaptopyruvate sulfotransferase; NADPH oxidase, nicotinamide adenine dinucleotide phosphate oxidase; NO**∙**, nitric oxide; eNOS, endothelial nitric oxide synthase; iNOS, inducible nitric oxide synthase; nNOS, neuronal nitric oxide synthase; ROS, reactive oxygen species.

During the last three decades, redox-mediated regulatory processes have been recognized as crucial signalling devices that have a high impact on cellular dynamics such as viability, proliferation, migration, cell contraction, and relaxation as well as gene expression under physiological and pathophysiological conditions. In particular, a massive synthesis of redox-active compounds such as reactive oxygen species (ROS) and nitric oxide (NO**∙**) can be observed in an inflammatory environment. Redox-active mediators are small inorganic molecules that are produced in a well-coordinated fashion in nearly all living beings. ROS such as superoxide (O_2_^−^), hydrogen peroxide (H_2_O_2_), the hydroxyl radical (HO∙), and hypochlorous acid (HOCl) are formed from molecular oxygen in the respiratory chain or by a series of O_2_-consuming enzymes such as the nicotinamide adenine dinucleotide phosphate (NADPH) oxidases or P450 oxidoreductases [1] (Figure 1A). Only the different subtypes of NADPH oxidases, i.e., NOX1-5, Duox1, and Duox2, will produce ROS as main reaction products that trigger important signalling processes, while most other ROS-producing mammalian enzymes produce it as unwanted and often noxious byproducts [2,3] or, as demonstrated for NOX2, potentially contribute to the innate immune defense by killing invading microorganisms or tumour cells [4,5]. ROS formation often occurs when an O_2_-consuming reaction proceeds under suboptimal conditions such as substrate or co-factor deficiency. This phenomenon is well characterized for nitric oxide synthases, especially the endothelial form (eNOS). Depletion of the essential cofactor BH_4_ shifts NO**∙** production to ROS formation, an issue called eNOS uncoupling that has an impact, especially in the cardiovascular system [6].

A further group of redox-active mediators consists of the small molecules NO**∙**, carbon monoxide (CO), and hydrogen sulfide (H_2_S). These molecules possess well-defined properties as signalling devices. Moreover, they readily diffuse through biological membranes and they are synthesized by various enzymes in a well-coordinated manner. Therefore, the members of these signalling factors are nowadays referred to as gasotransmitters [7]. In contrast to the production of ROS, the production of gasotransmitters is under tight control on the transcriptional and translational levels, and only a limited amount of gasotransmitter synthesizing enzymes control the gasotransmitter levels in the cellular environment. 

The so far best-characterised gasotransmitter regarding its production as well as action is NO**∙**. NO**∙** was found in 1987 as the long-sought “missing link” that drives signals from the endothelium to smooth muscle cells as an endothelium-derived relaxing factor (EDRF) [8,9]. Remarkably, besides this protective effect, NO**∙** possesses also noxious characteristics in mediating macrophage-derived cytotoxicity at high concentrations [10,11]. The reason for this contradictory behaviour is based on the ability of NO**∙** to form the potent oxidant peroxynitrite by the reaction with superoxide anions. To date, three different isoforms of nitric oxide synthases have been reported (Figure 1B). The existence of the neuronal form nNOS (also referred to as NOS1 or bNOS) is mainly expressed in neuronal cells located in the brain and spinal cord (cerebral cortex, cerebellum, hippocampus, and hypothalamus), but is also found among others in the glomerular macula densa and skeletal muscle [12,13]. eNOS (also known as NOS3) is mainly expressed in endothelial cells. nNOS and eNOS are regarded as the constitutive and Ca^2+^-dependent nitric oxide synthases meaning that their regulation occurs rather on the posttranslational level than on the transcriptional level. In contrast, the inducible nitric oxide synthase (iNOS) is transcriptionally regulated in an inflammatory environment after the challenge with inflammatory mediators such as bacterial lipopolysaccharides (LPS), interferon-γ (IFN-γ), interleukin-1β (IL-1β) or tumour necrosis factor α (TNF-α) [14,15]. iNOS is typically expressed in inflammatory cells such as macrophages and neutrophils, but also in tissue-resident cells, among others in glomerular mesangial cells and smooth muscle cells [16,17].

In addition to the nitric oxide synthases, NO**∙** can also be produced from nitrite by several nitrite-reducing enzymes that are metal-containing proteins and members of the eukaryotic molybdenum-dependent enzyme family [18].

Heme oxygenases (HO) convert heme to biliverdin, free ferrous iron (Fe^2+^) and CO. Two HO isoforms have so far been characterized (Figure 1C). The expression of the inducible form HO-1 is predominantly under the control of the transcription factors hypoxia-inducible factor (HIF) and nuclear factor erythroid 2-related factor 2 (Nrf2) and, consequently, HO-1 is most abundant under conditions of hypoxia or oxidative stress [19,20]. In contrast, HO-2 is constitutively expressed and serves as an oxygen sensor in the carotid body [21]. The existence of a third form of heme oxygenases (HO-3) is still a matter of debate. Most likely, HO-3 is a pseudogene that is not translated to a functional heme-degrading enzyme [22]. 

H_2_S, a poisonous gas that affects the respiratory chain has been recognized in 1996 as a gaseous neuromodulator that supports the activity of the glutamate NMDA receptor [23]. Similar to other gasotransmitters, H_2_S is endogenously produced by different enzymes in mammalian cells (Figure 1D). Cystathionine γ-lyase (CSE) and cystathionine β-synthase (CBS) are well-characterised enzymes of the transsulfuration pathway that produce H_2_S by a complex biochemical cascade using L-cysteine or L-homocysteine as substrates (for review see: [24,25]). In analogy to the enzymes involved in NO**∙** and CO synthesis, the regulation of CSE and CBS expression or activity occurs on different levels. In contrast to CBS, CSE is potently regulated on the transcriptional level, among others via the transcription factors nuclear factor κB (NFκB) and Nrf2 [26,27,28] and this strongly indicates a role for CSE-mediated H_2_S formation under inflammatory conditions and oxidative stress. 3-Mercaptopyruvate sulfotransferase (3-MST) is a predominantly mitochondrial enzyme and produces H_2_S using 3-mercaptopyruvate (3-MP) and pyruvate as substrates [29]. The rate-limiting substrate 3-MP for this biochemical reaction is provided by the activity of cysteine aminotransferase (CAT) or D-aminotransferase (DAO) an enzyme that converts D-cysteine from dietary uptake to 3-MP [30].

Taken together, ROS and the gasotransmitters NO**∙**, CO, and H_2_S are produced in a complex but well-coordinated manner in mammalian cells and the existence of at least two enzymes for each redox-mediator warrants their defined spatial and temporal formation that is needed to exert their specific biological effects. The immense research on ROS and gasotransmitter synthesis and action in the past two decades led to the conclusion that redox mediators mainly affect two target structures, namely metal centers of metalloproteins and, with the exception of CO, redox modifications of cysteine thiols, now referred to as thiol-based redox switches by sulfenylation (or the further oxidation levels of thiols by sulfinylation and sulfonylation) as well as nitrosation or sulfhydration [28,31] (Figure 1). It is important to note that ROS and gasotransmitters potently affect their own synthesis and this is best described by their action on thiols of redox-sensitive transcription factors, such as Nrf2 and NFκB, that in turn regulate the synthesis of gasotransmitters by the transcriptional activation of iNOS, HO-1 and CSE expression, resulting in a mutual machinery of gasotransmitter action and expression. Molecular oxygen (O_2_) is not produced in mammalian cells and this is most likely the reason to exclude it so far as a member of the gasotransmitter family. However, O_2_ is in analogy to gasotransmitters a versatile gas with specific signalling properties and it serves as the substrate for all ROS-producing enzymes. Moreover, O_2_ sensing by prolyl hydroxylases (PHDs) plays an important role in the transcriptional regulation of iNOS and HO-1 by the hypoxia-inducible transcription factors (HIF) 1 and 2. Therefore, we decided to include the effects of O_2_ in the following considerations regarding the interaction of redox and sphingolipid signalling.

### 1.2. Sphingolipid Biosynthesis, Degradation, and Signalling

**Figure 2:**
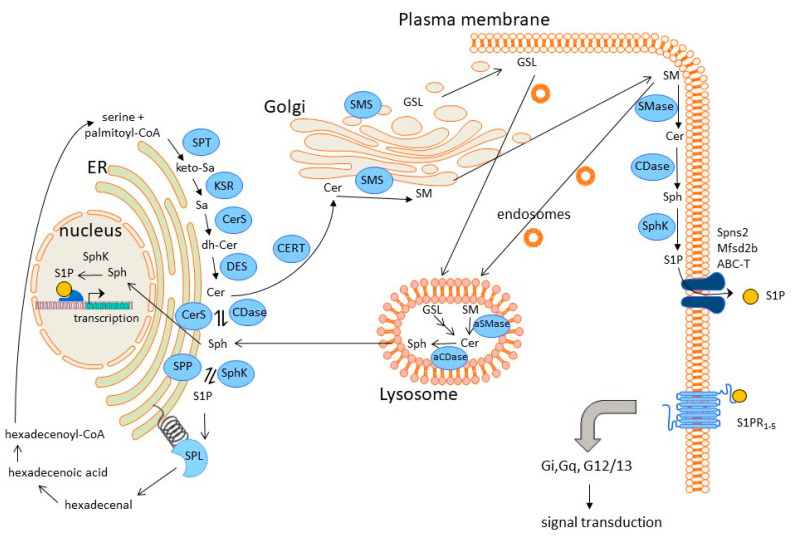
Subcellular pools of sphingolipids and their biosynthesis and degradation routes. Abbreviations: ABC-T, ATP-binding cassette transporter; CDase, ceramidase; Cer, ceramide; CerS, ceramide synthase; CERT, ceramide transport protein; DES, dihydroceramide desaturase; GSL, glycosphingolipids; KSR, 3-ketosphinganine reductase; Mfsd2b, major facilitator superfamily transporter 2b; S1P, sphingosine 1-phosphate; S1PR, S1P receptors; SM, sphingomyelin; SMase, sphingomyelinase; SMS, sphingomyelin synthase; Sph, sphingosine; Sphk, sphingosine kinase; SPL, S1P lyase; Spns2, spinster homology protein 2; SPP, S1P phosphatase; SPT, and serine palmitoyl transferase. This figure was created using the Motifolio PPT Drawing Toolkits (www.motifolio.com, accessed on 9 January 2023).

Over the last two decades, sphingolipids have taken a center stage in the field of lipid signalling research. Originally, they were discovered in 1884 by the German surgeon Johann Ludwig Wilhelm Thudichum as brain lipids with enigmatic Sphinx-like properties. The simplest molecule characterized was dubbed sphingosine. Sphingosine is an 18-carbon amino alcohol with an unsaturated hydrocarbon chain (2-amino-4-trans-octadecene-1,3-diol) and serves as the backbone for most of the complex glycosphingolipids. More than 400 species have been identified so far. The de-novo biosynthesis of all these sphingolipids starts in the endoplasmic reticulum (ER) by the condensation of L-serine and the fatty acid palmitoyl-CoA (C16) by the action of a serine palmitoyl transferase (SPT) yielding a long-chain base with a length of 18 carbon atoms, i.e., 3-keto-sphinganine (Figure 2). The SPT is a pyridoxal phosphate-dependent enzyme and consists of three subunits, i.e., the SPTLC1 (LCB1), the SPTLC2 (LCB2), and a third small subunit, either ssSPTa or ssSPTb [32], which is mandatory for full activity of SPT. Depending on the composition of the three subunits, the enzyme is able to condensate other fatty acids with serine, such as myristoyl-CoA (C14) or stearoyl-CoA (C18) yielding the more rare 3-keto-sphinganine variants of C16 or C20 chain lengths [32,33]. 

As the SPT-catalyzed condensation to 3-keto-sphinganine is the rate-limiting step in sphingolipid biosynthesis, the SPT represents a key point of regulation. Recently, small membrane-bound proteins of the ER, the mammalian orosomucoid-like proteins (ORMDLs), were described as negative regulators of SPT [34]. These ORMDLs can act as ceramide sensors. Thus, if ceramides are high in cells, ORMDLs bind these ceramides and thereby trigger a negative feedback signal to reduce SPT activity and de-novo sphingolipid synthesis. 

3-Keto-sphinganine is further reduced to sphinganine (dihydro-sphingosine) by the enzyme 3-ketosphinganine reductase (KSR) in an NADPH-dependent reaction [35]. Subsequently, sphinganine is acylated to dihydro-ceramides by various dihydroceramide synthases (CerS). These enzymes accept as substrate not only the saturated sphinganine but also the unsaturated sphingosine. So far, six different CerS have been identified that show organ-specific expression profiles, and differ in the acceptance of the fatty acid species for acylation [36]. The most abundant ceramides species found in most cells are the C16:0 > C24:0 > C24:1 > C22:0. Dihydroceramides are then desaturated by the enzyme dihydroceramide desaturase [37], and then transported from the ER to the Golgi by a ceramide transfer protein (CERT) [38], where it is further used for the buildup of sphingomyelin and complex glycosphingolipids.

Degradation of sphingolipids mainly takes place in the lysosomal compartment [39,40] (Figure 2). By endocytosis, plasma membrane sphingolipids are internalized and reach the lysosomes where either sphingomyelins are directly cleaved by an acid sphingomyelinase (aSMase) to yield ceramides, or glycosphingolipids are stepwise deglycosylated by various enzymes to yield ceramides. In a final step, ceramides are deacylated by the action of an acid ceramidase to form sphingosine, which as a final lysosomal degradation product can exit the lysosome and at the ER, be reutilized by CerSs to build ceramides. 

Alternatively, sphingosine can be phosphorylated to sphingosine 1-phosphate (S1P) by sphingosine kinases (Sphk). Two subtypes (Sphk1, Sphk2) exist with varying subcellular distributions and modes of regulation [41,42,43]. When located at the ER, Sphks will generate S1P as an intermediate which is further degraded by an S1P lyase to phosphoethanolamine and hexadecenal [44]. This reaction is irreversible and it is the only way to eliminate sphingolipids from the cell besides a possible secretion to the extracellular space. Sphks located at other subcellular sites, such as the plasma membrane and nucleus, generate S1P for signalling purposes that take place either intracellularly through still ill-defined targets, or extracellularly through G protein-coupled S1P receptors [45,46,47]. 

These receptors include five subtypes, denoted S1P_1-5_, with each receptor subtype coupling to more than one G protein thus providing a complex network of signal transduction that defines a cell’s and tissue’s response to increased or reduced S1P levels. Over the past years, these receptors have served as attractive and highly druggable targets for pharmaceutical companies, and several agonists, antagonists, functional antagonists, and biased agonists have been developed and taken to clinical trials with four of them reaching FDA approval for the treatment of autoimmune diseases, mainly multiple sclerosis [46].

## 2. Cross-Regulation of Redox and Sphingolipid Signalling

### 2.1. Hypoxia and Sphingolipid Signalling

As O_2_ is crucial for human life, the body is equipped with mechanisms to cope with hypoxia in order to keep homeostasis. Cells are able to detect and respond to hypoxia by upregulating the expression of specific genes, which allows for adapting to the hypoxia-induced stress condition. This involves the transcription factors HIFs of which three subtypes exist, i.e., HIF-1α, -2α, and -3α [48]. Under hypoxia, these factors accumulate and interact with the aryl hydrocarbon receptor nuclear translocator (ARNT/HIF-1β) to form a heterodimer that translocates to the nucleus and binds to so-called HIF-responsive elements (HREs) within promoter regions of hypoxia-regulated genes [49]. However, the actual hypoxia sensors are the enzymes prolyl hydroxylases (PHDs), which under normal conditions, require oxygen to catalyze hydroxylation of HIFs [50] which directs it for ubiquitination and degradation by the E3 ligase von Hippel Lindau protein (pVHL) [51]. Under hypoxic conditions, the hydroxylation of HIF by PHDs is prevented, thus allowing HIFs to accumulate and interact with ARNT/HIF1β and to translocate to the nucleus.

The first evidence for a cross-regulation of sphingolipid metabolism by hypoxia came from cell culture studies of rat oligodendrocytes [52]. The myelination of these cells is an important process requiring high metabolic turnover and energy input. Consequently, these cells are very sensitive to injury caused by energy impairment as it arises under hypoxic conditions. Remarkably, the sphingolipid galactosyl-ceramide is highly enriched in myelin constituting approx. 30% of total myelin lipids. When exposing rat neonatal oligodendrocytes to progressive hypoxia, selective inhibition of GalCer synthesis preceding cell injury occurred. Investigation of the mechanism of this early event suggested that hypoxia inhibited the transport of newly synthesized ceramide from its site of synthesis at the ER to its site of galactosylation in the Golgi, resulting in an accumulation of ceramide. The direct target of hypoxia was not further pinpointed. The authors argued that this early inhibition of GalCer synthesis by hypoxia may be a reason why myelination is so sensitive to hypoxia [52]. 

In cardiomyocytes, the exposure of cells to hypoxia/reoxygenation causes tissue injury and in parallel ceramide accumulation. This increase in ceramide was caused by the activation of neutral sphingomyelinase (nSMase) and mechanistically involved increased ROS formation and c-Jun kinase activation [53]. Additionally, in hepatocytes, hypoxia triggered the activation of the aSMase resulting in increased ceramide formation and apoptosis [54].

Furthermore, in isolated small pulmonary arteries, acute hypoxia-induced rapid vasoconstriction is considered an adaptive physiological mechanism to optimize blood oxygen saturation by increasing pulmonary vascular resistance in poorly aerated lung regions [55]. Also in this setting, the mechanism involved hypoxia-mediated ceramide accumulation due to nSMase activation in pulmonary artery smooth muscle cells [55].

Another hypoxia-induced adaptive response of vascular cells is their enhanced growth and proliferation. It was shown that this growth process is accompanied by decreased ceramide levels and an increased level of the mitogenic lipid S1P. Since S1P is generated from the precursors ceramide and sphingosine through the action of ceramidases and sphingosine kinases, this implies that the two enzyme classes can be activated or induced under hypoxia [56]. In adipocytes, the alkaline ceramidase 2 (Acer2) was indeed identified as a HIF-2α target gene [57]. Moreover, several studies have reported that Sphk1 or Sphk2 are HIF target genes. Notably, the human Sphk1 promoter contains several potential HRE sites [58,59]. Anelli et al. [58] identified especially the HRE at -1731bp of the human Sphk1 promoter as a functional HRE in the human glioma cell line U87MG. By treatment with cobalt chloride, which mimics hypoxic conditions, only HIF-2α increased its binding to the HRE and stimulated Sphk1 transcription. Different from this report, Schwalm et al. [59] used the immortalized human endothelial cell line EA.hy926 and identified, by HRE mutation and deletion studies, that the HRE at -1268 of the hSphk1 promoter was functional, and both HIF-1α and HIF-2α participated in the hypoxia-induced Sphk1 transcription. As different tumour cells are differentially equipped with Sphk1 and Sphk2, those cells with high levels of Sphk1 have low Sphk2 levels, and vice versa, it may well be possible that in certain tumour or non-tumour cell types, hypoxia could also affect Sphk2 expression. Indeed, in human pulmonary smooth muscle cells, acute hypoxia for 2 days increased mRNA of both Sphk1 and Sphk2, while chronic exposure to hypoxia for 14 days only showed increased Sphk1 mRNA [60]. The contribution of this intermediate increase of Sphk2 mRNA to lung pathology remains unclear. However, in the lung cancer cell line A549, hypoxia also increased Sphk2 protein expression and activity which provoked chemoresistance of the cancer cells [61]. Such a stimulating effect of hypoxia on Sphk2 was not detected in EA.hy 926 endothelial cells where hypoxia rather downregulated Sphk2 mRNA [59].

Increased Sphk1 activity under hypoxia was also observed in human prostate and brain cancer cell lines, although the effect was very transient with a maximal activation after 2 h of hypoxia with a rapid decline thereafter [62].

Another way of cross-regulation of HIF-1/2α and Sphk was recently reported by Hait et al. [63] showing a direct interaction of specifically Sphk2 with HIF-1/2α in protein complexes at the HRE sites of promoter regions of HIF target genes, such as VEGF, where it promoted histone H3 acetylation and gene transcription [63]. Furthermore, in hematopoietic stem cells, Sphk2 was shown to interact with both pVHL and PHD2 thereby facilitating HIF-1α ubiquitination and degradation independent of Sphk2 catalytic activity. Deletion of Sphk2 thus resulted in HIF-1α stabilisation, increased expression of the glycolysis checkpoint protein pyruvate dehydrogenase kinase 3 (PDK3), and consequently, an improved metabolic fitness of HSCs [64].

In various cancer cells, HIF-1α was shown to be stabilized by the Sphk1/S1P axis involving Sphk1 catalytic activity, S1P export, and autocrine action through S1P receptors [62]. Later on, similar data were reported for HIF-2α regulation by the same Sphk1/S1P axis in renal cancer cells [65]. In renal physiology, HIF-2α is the main transcription factor regulating erythropoietin (Epo) production which occurs in renal interstitial fibroblasts [66]. Such renal interstitial fibroblasts were isolated from *Sphk1^−/−^* and *Sphk2^−/−^* mice, and exposed to 1% O_2_. Data revealed that only the depletion of Sphk1 abrogated hypoxia-induced HIF-2α protein stability and Epo synthesis, while Sphk2 depletion rather upregulated HIF-2α protein levels and Epo synthesis [67]. This regulation of HIF-2α by Sphks may also have therapeutic utility as it proposes that Sphk2 inhibitors could have beneficial effects in patients suffering from chronic kidney disease and anemia, while Sphk1 inhibitors could be useful to treat diseases where erythropoietin synthesis is too high, such as secondary congenital erythrocytosis, that develops due to mutations in genes regulating Epo syntheses, such as *VHL*, *EGLN1*, *EPAS1*, or *EPO* [68].

Another sphingolipid-regulating gene, that is activated by hypoxia, is the serine palmitoyltransferase SPT2 [69]. In SH-SY5Y neuroblastoma cells, hypoxia enhanced cellular ceramide levels in parallel to undergoing increased apoptosis. Mechanistically, the increased ceramide formation was due to increased de-novo synthesis as the first enzyme in the de-novo pathway, i.e., SPT2 was transcriptionally upregulated resulting in increased protein and enzymatic activity [69]. Notably, blocking SPT2 by specific siRNA, also reduced hypoxia-induced apoptosis, thus, further confirming the critical contribution of ceramide to apoptosis. 

The importance of the cross-regulation of HIF signalling and sphingolipids also became visible in skin-specific ARNT/HIF-1β-deficient mice. These mice die shortly after birth by severe dehydration due to skin barrier defects and water loss [70]. Ceramides are key molecules regulating the epidermal permeability barrier and are highly enriched in the stratum corneum. Dysregulation or defects in the formation of extracellular ceramide structures is known to disturb barrier function [71]. Consequently, in ARNT/HIF-1β-deficient mice, sphingosine and ceramides were greatly reduced, while sphinganine was increased suggesting that ARNT/HIF-1β regulates an essential step in the ceramide biosynthetic pathway, such as the dihydroceramide desaturases (Des-1 and Des-2). Indeed, the mRNA expression of Des-2 was strongly reduced in keratinocytes of ARNT/HIF-1β-deficient epidermis [70]. 

ARNT/HIF-1β is also an essential binding partner of the aryl hydrocarbon receptor (AhR), which is a transcription factor activated by toxic environmental chemicals, such as dioxin-like polychlorinated biphenyls, or endogenously produced metabolic waste products such as indoxyl sulfates [72,73]. Thus, it is well possible that a cross-talk exists between environmental stress, hypoxia, and sphingolipids.

Recently, Majumder et al. showed that the AhR is a positive regulator of various genes in the sphingolipid biosynthesis pathway [74]. *Ahr*-deficient mice had reduced sphingolipid content in various tissues including the central nervous system. Specifically, the sciatic nerve had reduced ceramide levels and showed thinner myelin sheaths, and this correlated with a locomotor deficiency of the knockout mice [74,75]. These mice also had reduced plasma S1P levels suggesting that AhR may also be a regulator of the immune and vascular system [74]. All these data show that there is a mutual regulation of HIF transcription factors and sphingolipid metabolism which may have an impact on the cell’s adaptation to acute and chronic stress due to reduced oxygen levels.

### 2.2. Hyperoxia and Sphingolipid Signalling

Under certain conditions, cells and tissues may also be exposed to enhanced oxygen levels. For example, in a therapeutic setting of respiratory distress syndrome in adults, such as ARDS, or in preterm infants, mechanical ventilation and supplementation with O_2_ are used to treat and prevent hypoxia. However, severe or prolonged hyperoxia can lead to ROS formation and oxidative stress, which is detrimental to various organs, including the lung causing bronchopulmonary dysplasia [76]. Although bronchopulmonary dysplasia is a multifactorial disease, hallmark processes include inflammation, matrix remodelling, and apoptosis. As sphingolipids can principally regulate all these cellular processes, it seems obvious that sphingolipids contribute in one way or the other to disease pathogenesis.

In this view, it was shown that exposure of neonatal mice to hyperoxia enhanced S1P levels in lung tissues, and it was speculated that increased S1P signalling mediates neonatal lung injury. Only in *Sphk1*^−/−^ mice, but not in *Sphk2*^−/−^ or heterozygous *Sgpl1*^+/−^ mice, hyperoxia-induced lung injury was reduced [77]. Moreover, the Sphk1-selective inhibitor PF543 also ameliorated hyperoxia-induced lung injury thus strengthening the hypothesis that Sphk1 could be a useful therapeutic target of this disease [78]. Mechanistically, hyperoxia-induced lung injury involves increased mitochondrial ROS formation and this event is also reduced by Sphk1 blockade [78]. Notably, the Sphk1-driven S1P formation under hyperoxia depends on the cellular export of S1P from lung endothelial cells through the S1P transporter Spns2 and subsequent action through S1P_1_ and S1P_2_ receptors [79]. 

### 2.3. ROS and Sphingolipid Signalling

It is interesting to note that both hypoxia and hyperoxia conditions can lead to increased ROS formation. While hyperoxia-induced ROS formation is easily comprehensible, hypoxia-increased ROS formation seems more paradoxical. Still, it was shown that hypoxia causes increased ROS formation at the mitochondrial complex III, which then stabilizes HIF-1α [80,81], but may also act on multiple other enzymes including sphingolipid-metabolizing enzymes.

The first evidence for ROS involvement in ceramide formation came from cellular studies in the human leukemia cell line Molt-4 showing that a molecularly still undefined membrane-associated nSMase activity was inhibited by physiological concentrations of reduced glutathione (GSH) [82]. Conditions that deplete cellular GSH levels caused disinhibition of nSMase activity resulting in increased sphingomyelin hydrolysis and ceramide formation [82,83]. 

Meanwhile, multiple studies in different cell types have been performed to show that agents that cause oxidative stress and ROS formation lead to an activation of nSMase or aSMase resulting in increased ceramide formation and subsequent apoptosis (for review, see: [84,85,86,87]. Therefore, blocking ROS formation by antioxidants reduces ceramide formation and protects from apoptosis [88,89,90,91]. 

To date, the three nSMase genes, *Smpd2*, *Smpd3*, and *Smpd4*, encoding for the enzymes nSMase1, nSMase2, and nSMase3 have been cloned. These three subtypes show differential subcellular localisations and tissue expressions and may have distinct functions [92,93]. However, all of these enzymes turned out as redox-sensitive, although in a differential manner [94]. 

nSMase1 shows enhanced activity in the presence of reducing agents, such as DTT and β-mercaptoethanol, since cysteines participate in disulfide bridge formation and stabilisation [95]. Consequently, oxidized glutathione, H_2_O_2_, or peroxynitrite can inhibit the enzyme reversibly or irreversibly [96,97]. 

Sequence and mutational analysis of nSMase2 revealed several oxidant-sensitive cysteine residues in the C-terminal domain of nSMase2 [98]. These cysteines are involved not only in catalytic activity but also in the oligomerisation of the enzyme. The highest activity was found for the monomeric form, while oligomers exert reduced activity [98]. When mutating Cys^617^ to Ser, this keeps the enzyme in a monomeric form and is associated with increased activity. Moreover, generating a cellular system that lacked the thioredoxin antioxidant system, led to increased oligomer formation and reduced enzyme activity [98]. 

The nSMase3 was first isolated and purified from bovine brain and in vitro activity assays revealed that this subtype was efficiently inhibited by GSH [99]. The human homolog of nSMase3 is highly expressed in heart and skeletal muscle [100]. It shows no significant sequence homology to nSMase1 or nSMase2 but had some biochemical properties similar to nSMase2. The cellular activity was transiently enhanced by TNFα, but its direct inhibition by GSH was not further confirmed [100]. Rather, in myotubes, it was reported that nSMase3 by itself participates in ROS formation [101].

The *Smpd1* gene encodes for the aSMase that requires a pH of 4.5 for optimal activity and therefore is mainly localized in the lysosomes, but can also be secreted. This enzyme is also regulated by redox mechanisms [84]. Especially the C-terminal cysteine residue (Cys^629^) of aSMase is considered redox-sensitive, and a variety of chemical modifications of this residue, or mutation to Ser, resulted in enzyme activation [102]. Vice versa, the presence of DTT inhibited aSMase activity in vitro [103]. It was proposed that the thiol group stabilizes the inactive conformation of aSMase and by deprotonation of the thiol, aSMase is released from the inactive to an active conformation [94].

While all these studies support a signal flow from ROS to increased ceramide formation, other studies show that the opposite direction of signalling, i.e., from ceramide to increased ROS formation, is also existing, and this may lead to a self-accelerating loop of ceramide and ROS formation and consequently cell damage and death. Various mechanisms have been forwarded by which ceramide can cause mitochondrial dysfunction and ROS formation. Especially long-chain ceramide (C16) was shown to inhibit the mitochondrial respiratory chain (MRC) by directly inhibiting complexes I, III, and/or IV [104,105]. A reduced electron transport through the MRC is known to increase ROS generation [106,107]. Ceramide can also directly form channels in the mitochondrial outer membrane to allow cytochrome C release and caspase activation leading to apoptosis [108,109]. Alternatively, ceramide can directly interact with mitochondrial proteins, such as the pro-apoptotic Bax protein, and in this hybrid form to constitute channels in the mitochondrial membrane [110]. Furthermore, the direct binding of ceramide to the mitochondrial voltage-dependent anion channel 2 (VDAC2) has been shown [111], which serves as a stabilizing platform for Bax at the mitochondrial membrane and prevents its retrotranslocation to the cytosol [112]. 

Notably, the increase of the ceramide-ROS cycle and subsequent apoptosis has also been stressed as an effective novel therapeutic approach for cancer treatment. Especially the acid, neutral and alkaline ceramidases could serve as useful targets for intervention. Thus, in various cancer cell lines, the catalytic inhibition of these enzymes or their downregulation by RNAi resulted in enhanced ceramide, ROS, and cancer cell death [113,114,115,116,117].

In certain settings, the cell death triggered by the ceramide-ROS cycle can be reduced by the counter-molecule S1P, which then promotes cell survival. Therefore, activating the enzymes that convert ceramide to S1P, i.e., ceramidases and Sphks, will reduce the efficiency of anti-cancer treatment and predicts poorer survival of patients. On the other side, this escape from the ceramide-ROS cycle may be beneficial in pathologies where apoptosis is unwanted such as in systemic inflammation (sepsis) and ischemia-reperfusion injuries in various organs [87,118,119]. In line with such a protective and survival effect of S1P, the use of a potentially interesting new drug, the Sphk1 activator K6PC-5 revealed that intracellular enhancement of S1P inhibited oxygen-glucose deprivation/reoxygenation-induced apoptosis of myocardial cells in parallel to reducing ceramide and ROS [120].

As a further mechanism of escalation of ischemia-reperfusion injury, as shown in vivo in a cardiac I/R injury model and cardiomyocytes, ROS may, in addition to increase ceramide formation, also downregulate Sphk1 activity and S1P levels and thereby move the ceramide/S1P rheostat balance even further towards ceramide and apoptosis [121]. In this study, H_2_O_2_ was used to show that not only Sphk1 activity is inhibited, but also Sphk1 protein is downregulated, and the reaction was reversed by antioxidants. The mechanism of the Sphk1 protein downregulation remains open but it resembles the effect seen with other catalytic Sphk1 inhibitors which also induce Sphk1 degradation upon inhibition [122,123]. 

The involvement of S1P in ROS production is still controversial and different hypotheses have been forwarded. In this view, Keller et al. [124] showed that in a model of cannulated hamster resistance arteries, pressure-induced myogenic vasoconstriction through the activation of Sphk1/S1P and enhanced ROS formation. In isolated vessels, exogenous S1P also stimulates ROS formation, which mediates increased Ca^2+^ sensitivity necessary for full myogenic vasoconstriction [124]. Similarly, in the setting of hyperoxia-induced lung injury in neonates, S1P, produced by Sphk1 and exported by Spns2, acted through S1P receptors on lung endothelial cells to activate NADPH oxidase and ROS formation finally leading to lung injury [79]. In a corresponding mouse model of hyperoxia-induced lung injury, *Sphk1* depletion prevented lung injury, while a partial depletion of the S1P degrading S1P lyase (*Sgpl1^+/−^*) accelerated lung injury [79]. 

The pathophysiological contribution of the S1P-ROS signalling is also substantiated in a mouse model of cardiac fibrosis. By using Sphk1 transgenic mice, it was shown that high expression levels of Sphk1 led to progressive myocardial degeneration and fibrosis, with elevated levels of oxidative stress markers, and treatment of these mice with an antioxidant not only reduced ROS, but also cardiac fibrosis [125].

Altogether, these data highlight that there is a complex and still ill-defined crosstalk between ceramide, S1P, and ROS, which may also be cell-type and tissue specific.

### 2.4. Cross-Talk of Gasotransmitters and Sphingolipid Signalling

#### 2.4.1. Nitric Oxide (NO∙)

The role of NO**∙** as a signalling molecule has been extensively studied in the past [126]. The first direct target of NO**∙** to be identified was the hemoprotein soluble guanylate cyclase (sGC) [127]. NO**∙** binds to the heme iron of sGC forming a heme-nitrosyl complex and enzyme activation leading to the formation of cGMP, which in the endothelium, is a highly potent vasodilator [128]. 

As well as binding to metal ions, NO**∙** can also covalently bind to cysteines and tyrosines to form nitrosocysteine- and nitrotyrosine-modified proteins, which may cause a change in enzyme activity and function. By using the novel and sensitive methodology of mass spectrometry, multiple new proteins were identified to be modified by NO**∙** causing dysfunctional signalling and contributing to diseases including cancer and inflammation [129,130,131,132].

Interestingly, when analysing pituitary adenoma tissue in a nitrosoproteome approach, the S1P lyase (SPL, *Sgpl1*) was identified as tyrosine nitrated on two residues, Tyr^356^ and Tyr^366^ [131]. Since these two sites are within the catalytic domain of Sgpl1, NO**∙** modification might affect its catalytic activity, although this has not been proven yet. 

Another direct target of NO**∙** and a key signalling factor is the small G protein p21^ras^, which is cysteine nitrosated in the presence of a NO**∙** donor resulting in more active p21^ras^ and downstream signalling, such as NFκB activation [133] and mitogen- and stress-activated protein kinase SAPK activation [134,135,136,137]. 

By affecting these fundamental signalling cascades, which in turn regulate many transcription factors, NO**∙** can interfere with gene transcription of many genes, including sphingolipid-regulating enzymes, and thereby alter cell responses. Indeed, NO**∙** was shown to affect sphingolipid signalling in different cell types. Thus, treatment of renal mesangial cells [138] or endothelial cells [139] with NO**∙** donors caused a concentration-dependent increase in cellular ceramide formation. This effect mechanistically involved upregulated activities of both aSMase and nSMase, and may occur in a similar manner as reported for ROS-activated SMase activity [94], i.e., directly by cysteine modification, or indirectly by GSH depletion. 

In contrast to these studies, Falcone et al. [140] rather suggested an inhibitory effect of NO**∙** on aSMase. They showed that apoptosis, induced either in dendritic cells by LPS [140], or in monocytic U937 cells with TNFα [141,142], involves aSMase activation and ceramide formation. This apoptotic effect of LPS and TNFα was blocked by NO**∙**, cGMP, and cGMP-dependent protein kinase (PKG, cGK) activation [140,141,142]. Such an anti-apoptotic effect of NO**∙** through aSMase inhibition in tumour-associated macrophages seems also to be relevant in cancer therapy where it leads to chemoresistance [143]. However, the detailed mechanism of aSMase inhibition by PKG is still not clear, but at some level must involve a phosphorylation step by PKG. While many substrates of PKG have been described [144], aSMase has so far not been confirmed as a direct substrate of PKG. Therefore, phosphorylation of an upstream factor responsible for aSMase inhibition seems likely.

Another level of regulation of aSMase by NO**∙** may exist by protein-protein interactions through nitrosated residues. In this regard, it was shown that nitrosylation of procaspase-3 promoted the direct interaction of procaspase-3 with aSMase, and this interaction had an inhibitory effect on procaspase-3 function thus inhibiting downstream caspase-8 activation and thereby reducing cell death [145].

Clearly, there are more factors than the SMases that determine whether cellular ceramides increase and especially the balance between SMases and CDase activities is crucial as well. Therefore, in situations when both activities of SMase and CDase increase, ceramide may not accumulate. This situation was shown for mesangial cells. When exposed to the pro-inflammatory cytokines IL-1β and TNFα, SMase and CDase activities increased in parallel thus resulting in a net unaltered ceramide [138,146]. In the case of NO**∙**, ceramide accumulates because SMase activities are increased while CDase activities are reduced [138,147]. This was mechanistically further approached and it seems that NO**∙** causes proteasomal degradation of neutral CDase [148]. 

#### 2.4.2. Carbon Monoxide (CO)

CO is structurally very similar to NO**∙**, but it is not a radical and therefore is more stable than NO**∙** and diffuses freely. Endogenously, it is mainly generated as a side product of heme degradation to bilirubin by the catalytic action of heme oxygenases [149,150]. Consequently, most of the CO derives from hemoglobin and there is a constant generation of this small molecule as a result of red blood cell turnover. In addition, a small part also derives from other heme-containing proteins (hemoproteins) such as myoglobin, cytochrome c oxidase, cytochrome P450, and even nitric oxide synthases [150,151]. CO produced by nitric oxide synthases is thought to regulate neurotransmission and blood flow in the central nervous system.

In biological systems, CO reacts with reduced transition metals such as iron in hemoproteins [151]. High concentrations of CO are toxic and this is due to its binding to hemoglobin which occurs with many-fold higher affinity than oxygen. Consequently, CO displaces oxygen from the heme-binding resulting in impaired respiration and tissue hypoxia [151]. However, it is now appreciated that low concentrations of CO, as it steadily arises from red blood cell turnover, are cytoprotective. This has led to the development of CO-releasing molecules (CORM) for various therapeutic purposes [152] such as regulation of vascular tone, platelet aggregation, and inflammation. Mechanisms that mediate this protective effect of CO include the direct binding of CO to the various hemoproteins including sGC, cytochrome P450 proteins, cytochrome c oxidase, NADPH oxidase, and nitric oxide synthase [153]. Indeed, it was shown that CO and cigarette smoke, similar to NO**∙**, can activate the sGC yielding increased cGMP levels and endothelial relaxation [154,155,156]. 

The influence of CO on sphingolipids has only been poorly studied over the years. In one early case report of CO poisoning causing a myelinopathy, manifested by demyelination of neuronal cells, lipid analysis in the brain revealed decreased levels of total phospholipids, cerebrosides, sphingomyelins, and free cholesterol with enhanced cholesterol esters in the white matter. No changes occurred in the grey matter of the cerebral cortex [157]. Similarly, in a rat model of experimental acute CO poisoning, decreased brain gangliosides were detected together with changes in myelin [158].

Multiple stimuli can induce HO-1 expression and thereby also generate CO [153]. Among those, S1P was identified as an inducer of HO-1 in primary human macrophages [159]. This effect was mediated by the S1P_1_ receptor and resulted in a polarisation of macrophages to an M2 phenotype and an anti-inflammatory and anti-apoptotic reaction [159]. However, in other cells, such as in human leukemia cells, S1P and its acylated form, ceramide 1-phosphate (C1P), both downregulated HO-1 which was proposed to be an important mechanism in the pro-metastatic effect of these lipids on leukemia cells [160]. Moreover, short-chain C2-ceramide induced HO-1 in rat primary astrocytes [161]. This occurred through the activation of the AMPK and MAPK signalling cascades.

These few data vaguely propose that there may exist a bidirectional regulation of the sphingolipid rheostat and HO-1/CO, although a therapeutic use of this regulatory setting is not yet clear.

#### 2.4.3. Hydrogen Disulfide

To date, only few data are available to show a direct link between H_2_S and sphingolipids. Interestingly, among the three H_2_S-producing enzymes, only CBS is converting L-cysteine to L-serine and H_2_S. L-serine is also the precursor in the de-novo biosynthesis of sphingolipids, and is directly used by the SPT for condensation with palmitoyl-CoA. Thus, changing the CBS expression or activity may have an impact on sphingolipid synthesis. In a recent study on isolated mouse aorta rings, it was shown that L-cysteine and L-serine have a vasorelaxant effect [162]. The vascular effect of both L-cysteine and L-serine was reduced by a NOS inhibitor, but also by the SPT inhibitor myriocin and the S1P_1_ receptor antagonist W146, suggesting the involvement of both NO**∙** and S1P in the relaxant effect. It was speculated that this mechanism could be involved in the marked dysregulation of vascular tone in hyperhomocysteinemic patients (CBS deficiency) and may represent a feasible therapeutic target [162]. 

Since there is a well-studied bidirectional cross-talk between NO**∙** and H_2_S [162,163,164], and in view of the cross-regulation of NO**∙** on sphingolipid signalling (see Section 2.4.1), it seems very obvious that there is also a cross-talk between H_2_S-generating enzymes and sphingolipids.

In multiple myeloma cells, H_2_S donors synergistically enhanced apoptosis of cells induced by the green tea polyphenol (-)-epigallocatechin-3-O-gallate (EGCG), and thereby, potentiated the anti-cancer effect of EGCG in a mouse xenograft model [165]. This study further showed that in the presence of H_2_S donors, EGCG enhanced acid sphingomyelinase activity which was not seen by either substance alone. It was speculated that this effect on aSMase resulting in enhanced ceramide formation, is responsible for the increased apoptosis of cells [165].

Another interesting study showed that in human fibroblasts undergoing AKT-induced senescence, the expression of the enzyme cystathionine-β-synthase (CBS) was enhanced leading to increased H_2_S and GSH production, and consequently protected senescent cells from oxidative stress-induced cell death [166]. Especially in view of the fact that GSH is an endogenous inhibitor of nSMases, it could even be speculated that the protective effect of CBS on cell death, besides a direct protective effect of H_2_S, is additionally mediated by reduced ceramide formation through blocked nSMase activity.

**Figure 3:**
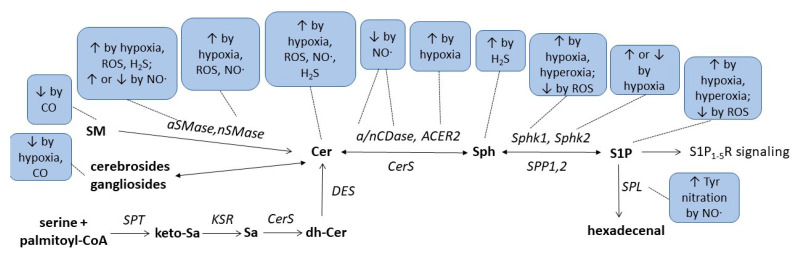
Summarizing scheme of gasotransmitters, hypoxia, and hyperoxia effects on sphingolipid metabolism. For abbreviations, see text.

Finally, a recent study suggested an indirect link between H_2_S and sphingolipids in a rat model of cerebral ischemia. In that study, the authors orally applied berberine to ischemic rats which improved the neuroinflammation and disease scores in tMCAO-induced cerebral ischemia [167]. It was shown that oral berberine acted on the gut microbiota and stimulated H_2_S production in the intestine, which subsequently activated the vagus nerve to subsequently alter the cerebral microenvironment resulting in less microglia activation and reduced neuroinflammation. Metabolomics analysis of various brain regions revealed changes in sphingolipid metabolism which may mediate the neuroprotection following vagus nerve activation. Notably, sphingosine was strongly increased in the ischemic rat cortex and downregulated by berberine treatment [167]. These data suggest that microbiota H_2_S production affects cerebral sphingolipid metabolism through vagus nerve activation. Altogether, the critical issues regarding the complex interaction of redox- and lipid-signalling described in this review article are summarized in Figure 3 and Table 1.

## 3. Perspectives

This review has focused on a topic, that has had a surprisingly long period of not being in the mainstream of pharmacology and drug development, but which the authors feel highlights an area of medicine that promises to become highly relevant for the translation and discovery of innovative new approaches to prevention, diagnosis, and treatments. The potential contribution of redox and lipid signalling to cellular regulation has been studied for decades and these elegantly orchestrated cellular signalling types of machinery have provided hallmarks of drug development, such as cyclooxygenase inhibitors and sphingosine 1-phosphate receptor modulators, just to name two eminent success stories in this field of research. Looking back over the last decades, one is struck by the extent of progress made in basic knowledge of redox biology and lipid biology and the corresponding translation into molecular and clinical medicine. However, as we would wish to emphasize, particularly the sophisticated interconnection of both signalling pathways is yet poorly understood and the novel ideas arising from this approach eventually will provide further hallmarks in the future of clinical and medical applications. As the content of this review highlights, scientists at the interface of two signalling devices have been instrumental in constituting new routes to drug development. The future prospects have to focus on optimizing the good, avoiding the bad, and unravelling new avenues of successful treatment of diseases.

## Figures and Tables

**Table 1:** Regulation of sphingolipids and their key enzymes by redox-active mediators.

Condition/ Enzyme	Hypoxia	Hyperoxia	ROS	NO∙	CO	H_2_S
Ceramides	↑OL [52], CM [53], HC [54], PA [55] ↓VSMC [56]		↑cancer cells [82,83,89], EC [90,91], MC [91], CM [53]	↑MC [91,138], EC [91,139] ↓DC [140], U937 [141,142]		↑cancer cells [165]
Sphingosine						↑cortex [167]
S1P	↑VSMC [56], EC [59], cancer cells [58,61]	↑mouse lung [77], human lung [78], EC [77]	↓CM [121]			
SM, Gangliosides					↓brain [157,158]	
Cholesterolesters					↑brain [157]	
Cerebrosides	↓OL [52]				↓brain [157]	
nSMase	↑CM [53], PA [55]		↑EC [90], CM [53]	↑MC [138]		
aSMase	↑HC [54]		↑EC [90]	↑MC [138] ↓DC [140], U937 [141,142]		↑cancer cells [165]
nCDase, aCDase				↓MC [138,147]		
ACER2	↑adipocytes [57]					
Sphk1	↑EC [59], PSMC [60], cancer cells [58,62]	↑mouse lung [77,79]	↓CM [121]			
Sphk2	↑cancer cells [61], PSMC [60] ↓EC [59]					
SPL				Tyr nitration [131]		
SPT2	↑neuroblastoma cells [69]					

Abbreviations: ACER2, alkaline ceramidase 2; aSMase, acid sphingomyelinase; nCDase, neutral ceramidase; Sphk1, sphingosine kinase 1; Sphk2, sphingosine kinase 2; SPL, sphingosine 1-phosphate lyase; SPT2, serine palmitoyltransferase 2; ROS, reactive oxygen species; NO**∙**, nitric oxide; CO, carbon monoxide; H_2_S, hydrogen sulfide; EC, endothelial cells; MC, mesangial cells; SM, sphingomyelins; OL, oligodendrocytes; CM, cardiomyocytes; DC, dendritic cells; PA, pulmonary arteries; U937, pro-monocytic human myeloid leukemia cell line; VSMC vascular smooth muscle cell; PSMC, pulmonary smooth muscle cells; ↑, upregulated; ↓, downregulated.
